# Supplementary material for: Disruption of the Mammalian Ccr4–Not Complex Contributes to Transcription-Mediated Genome Instability
Source: Cells. 2023 Jul 17;12(14):1868. doi: 10.3390/cells12141868 (PMC10378556; doi:10.3390/cells12141868)

Supplementary Figure S1

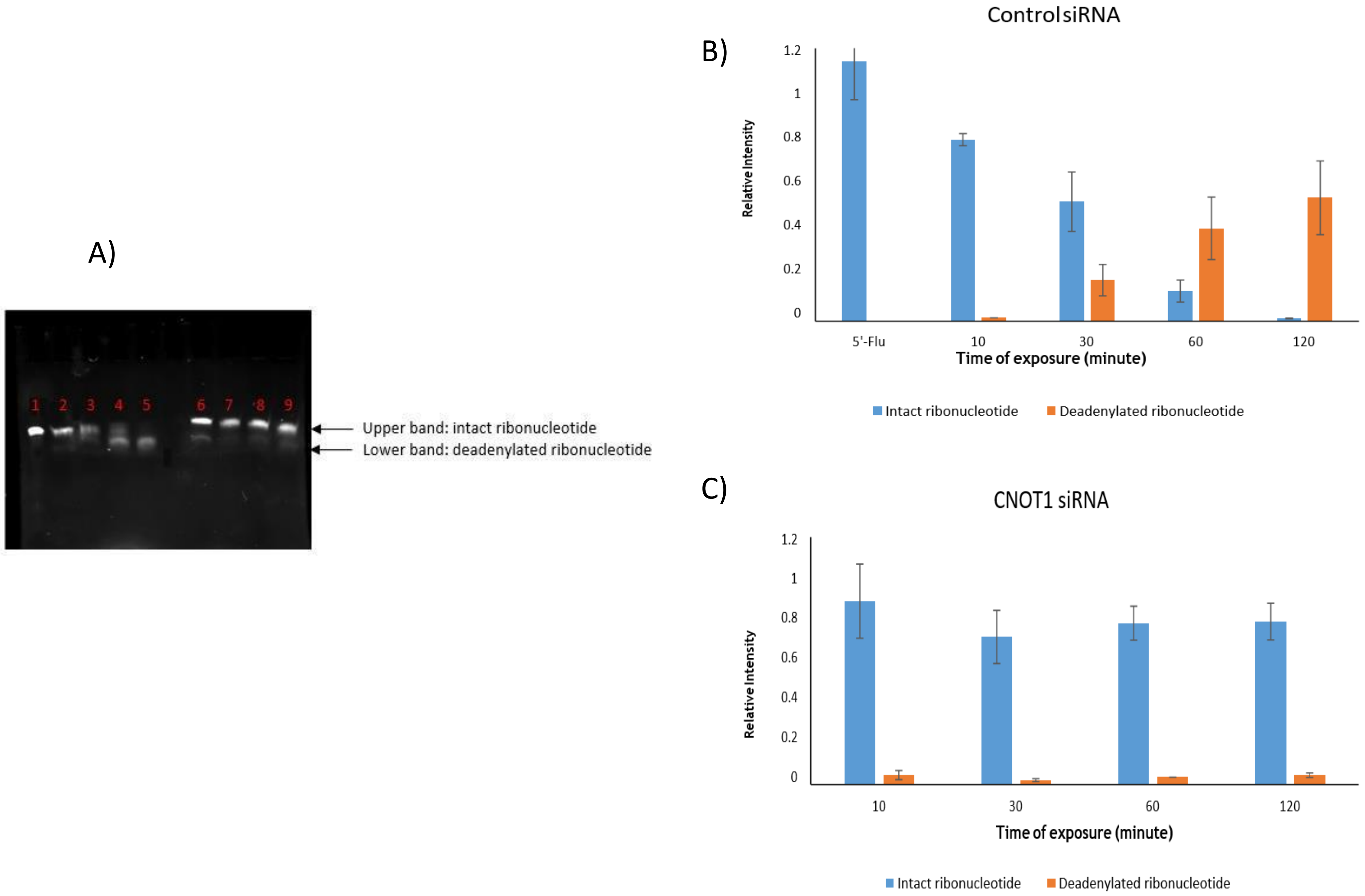

Supplementary Figure S2

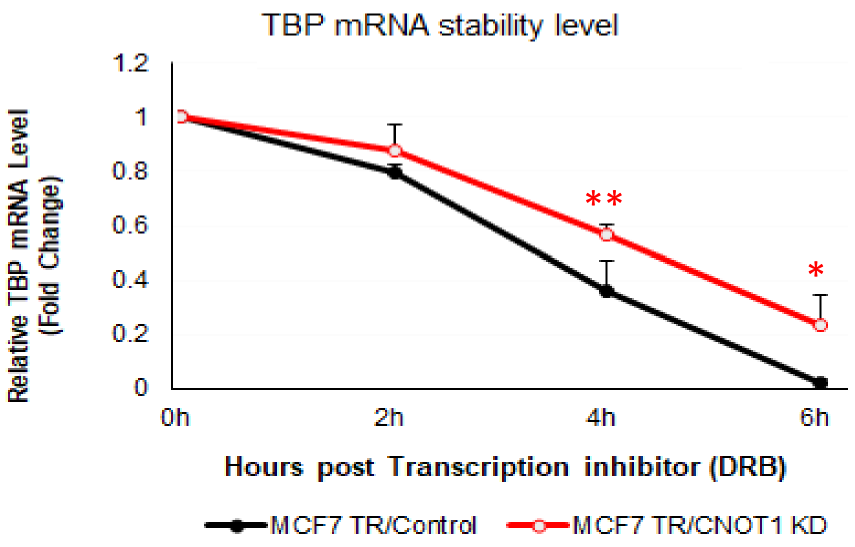

Supplementary Figure S3

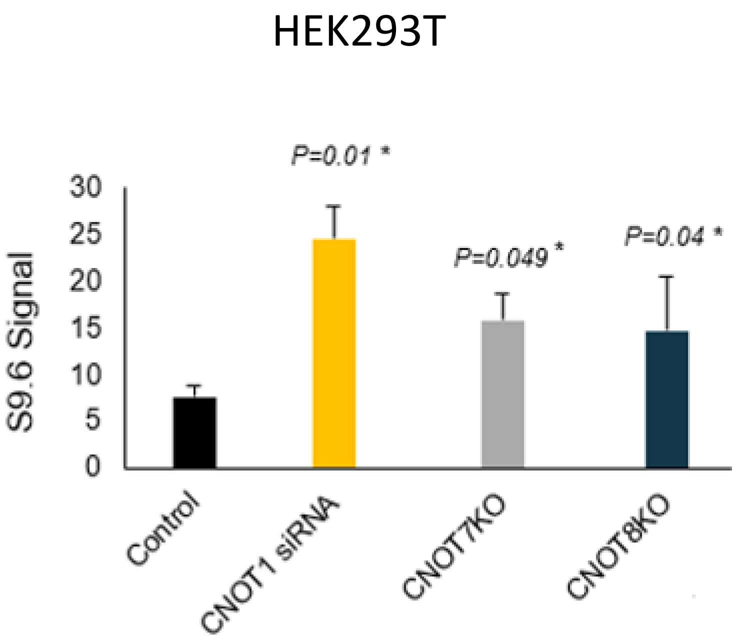

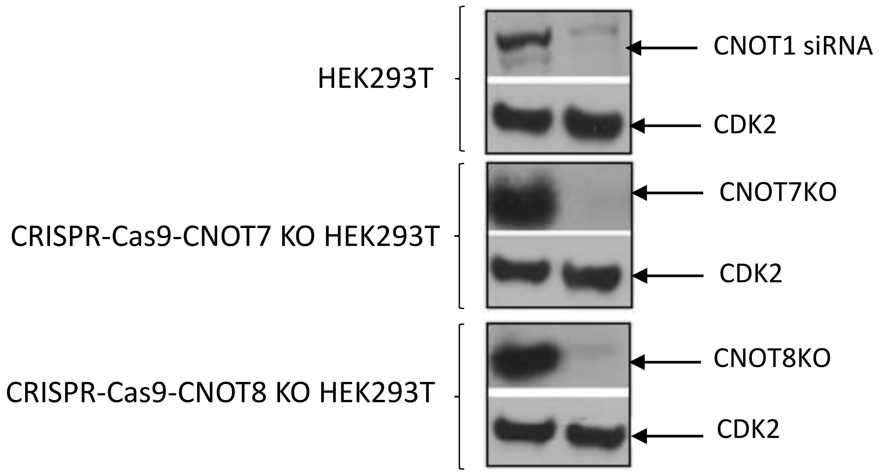

Supplementary Figure S4

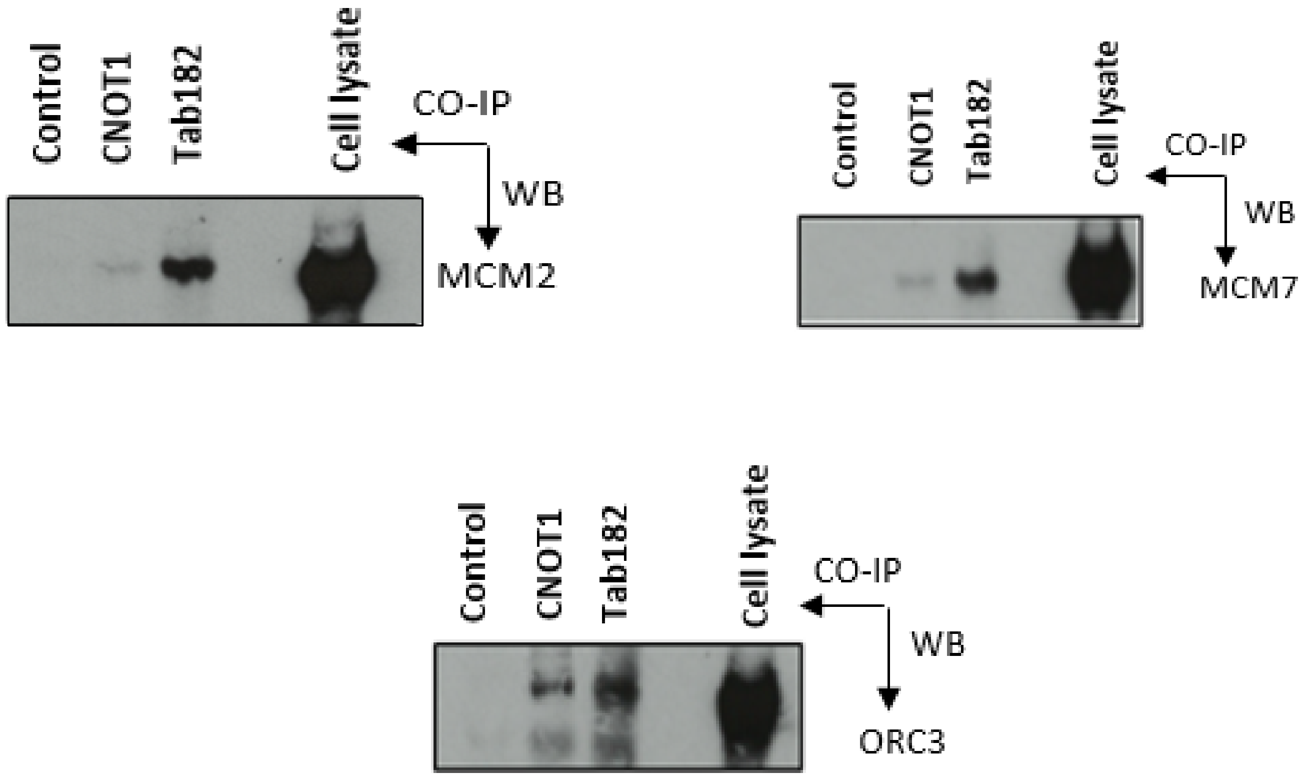

Supplementary Figure S5

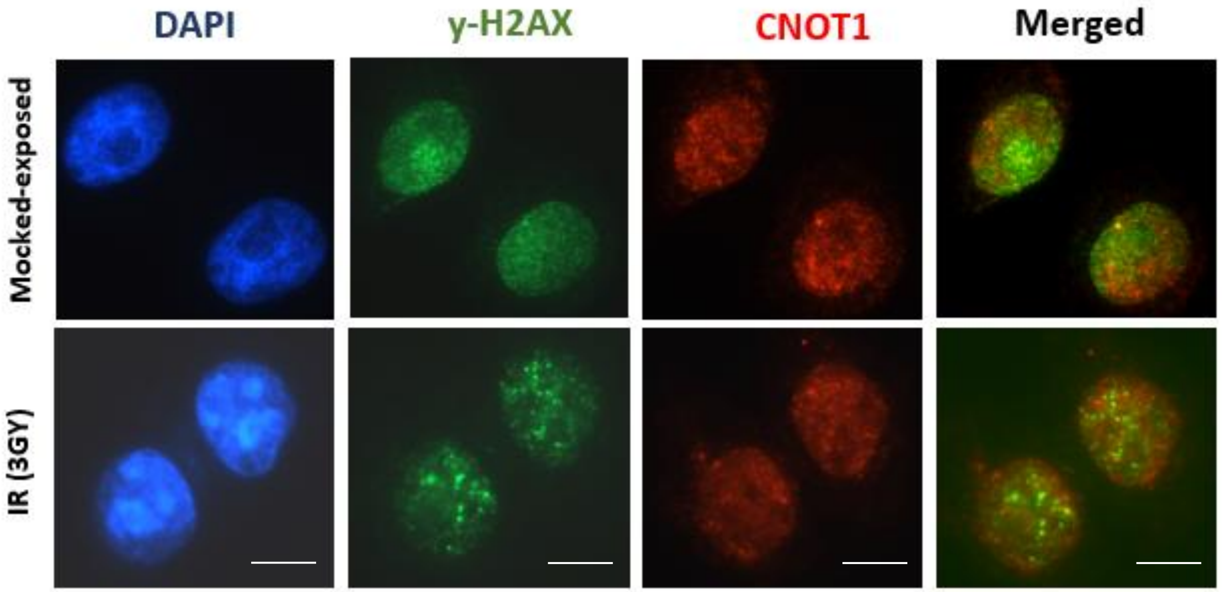

Supplementary Figure S6

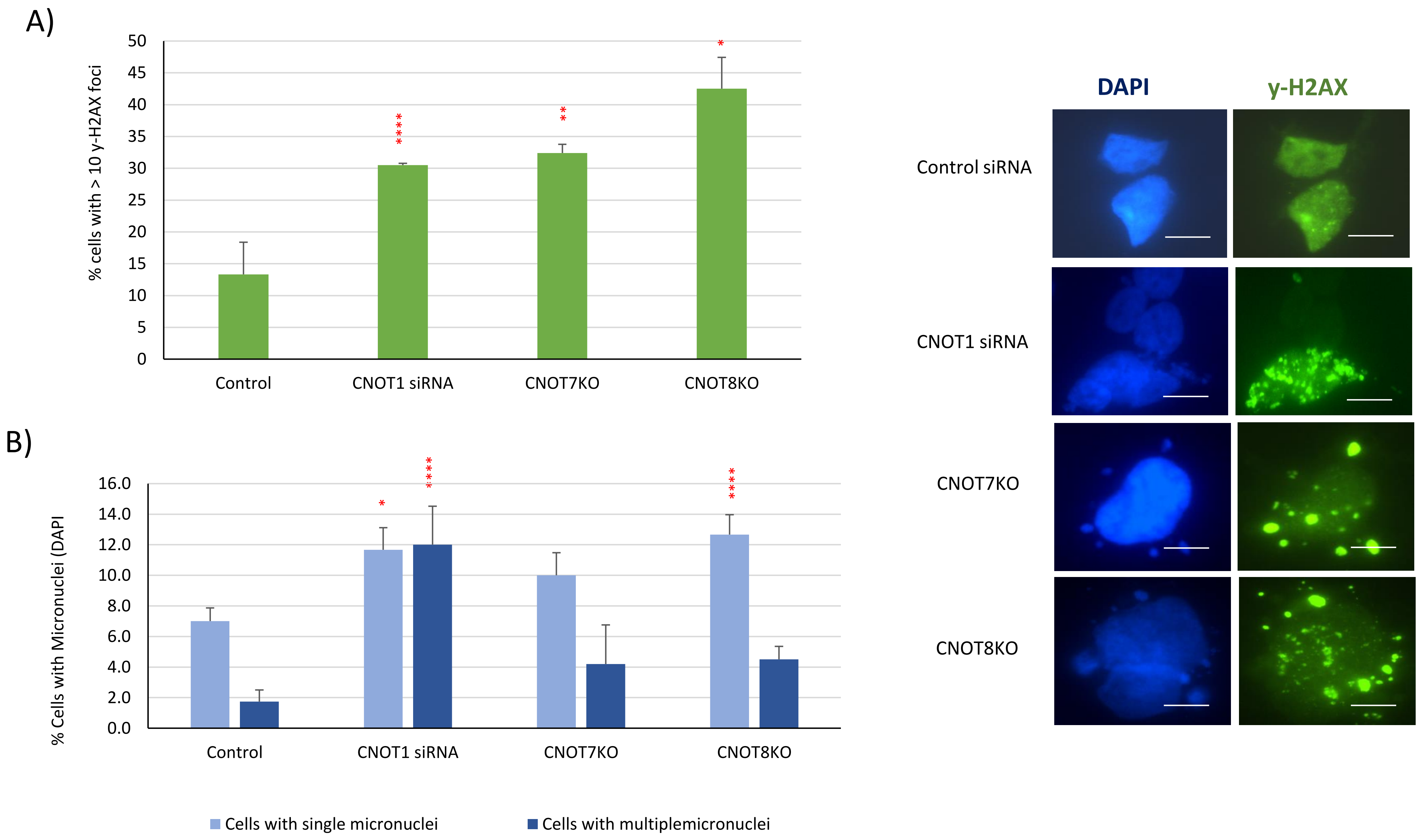

Supplementary Figure S6

C)

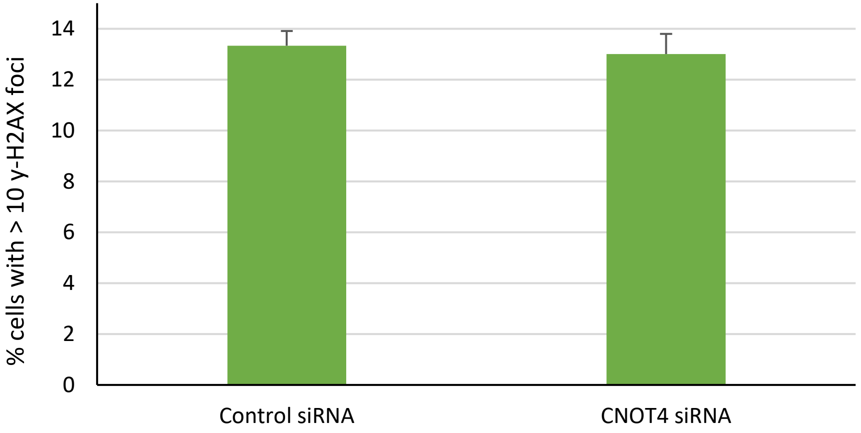

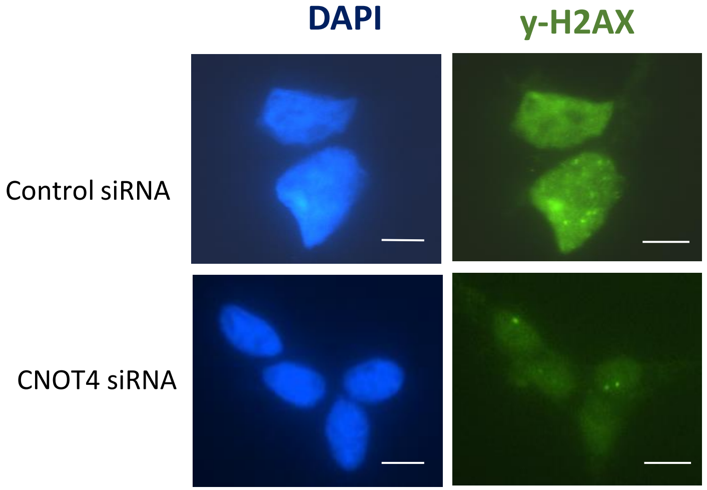

D)

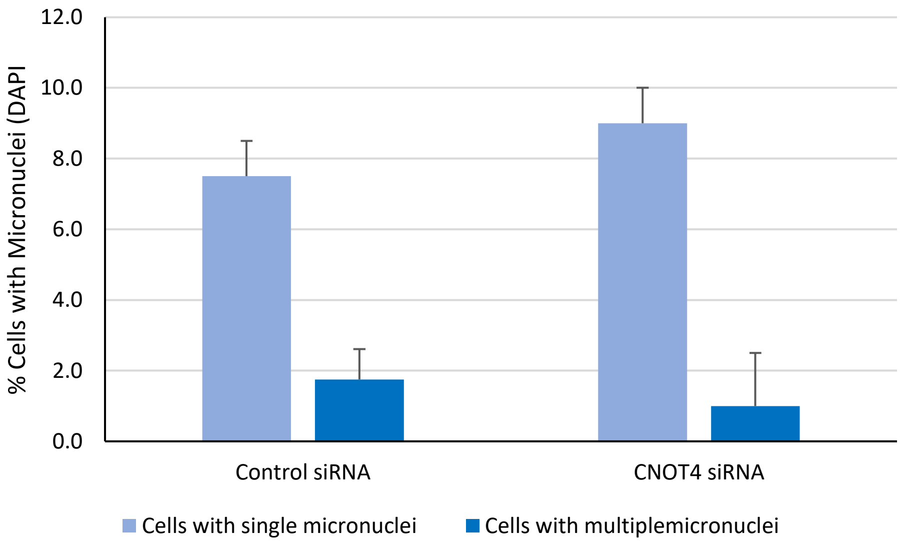

Supplementary Figure S7

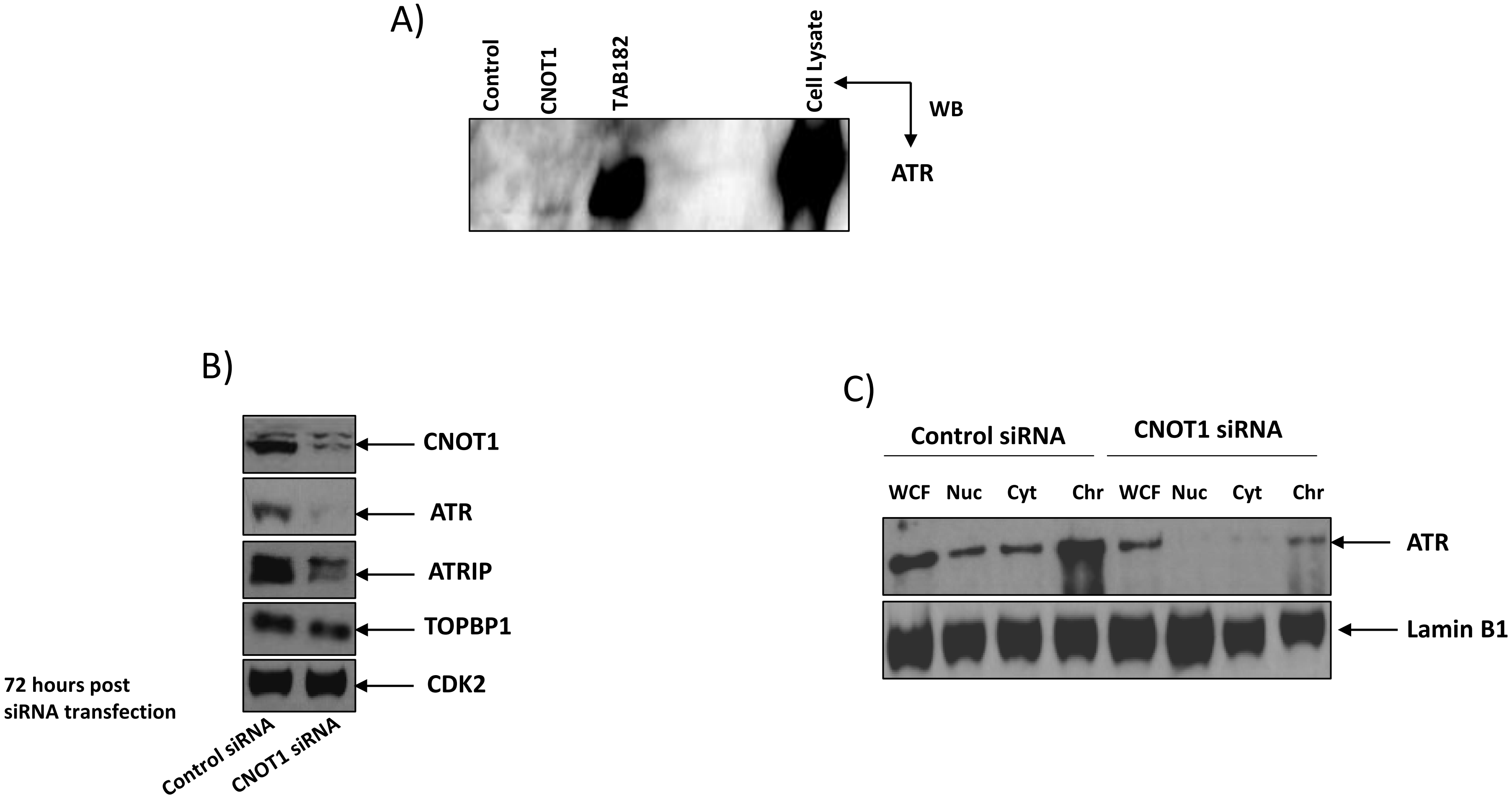

Supplementary Figure S8

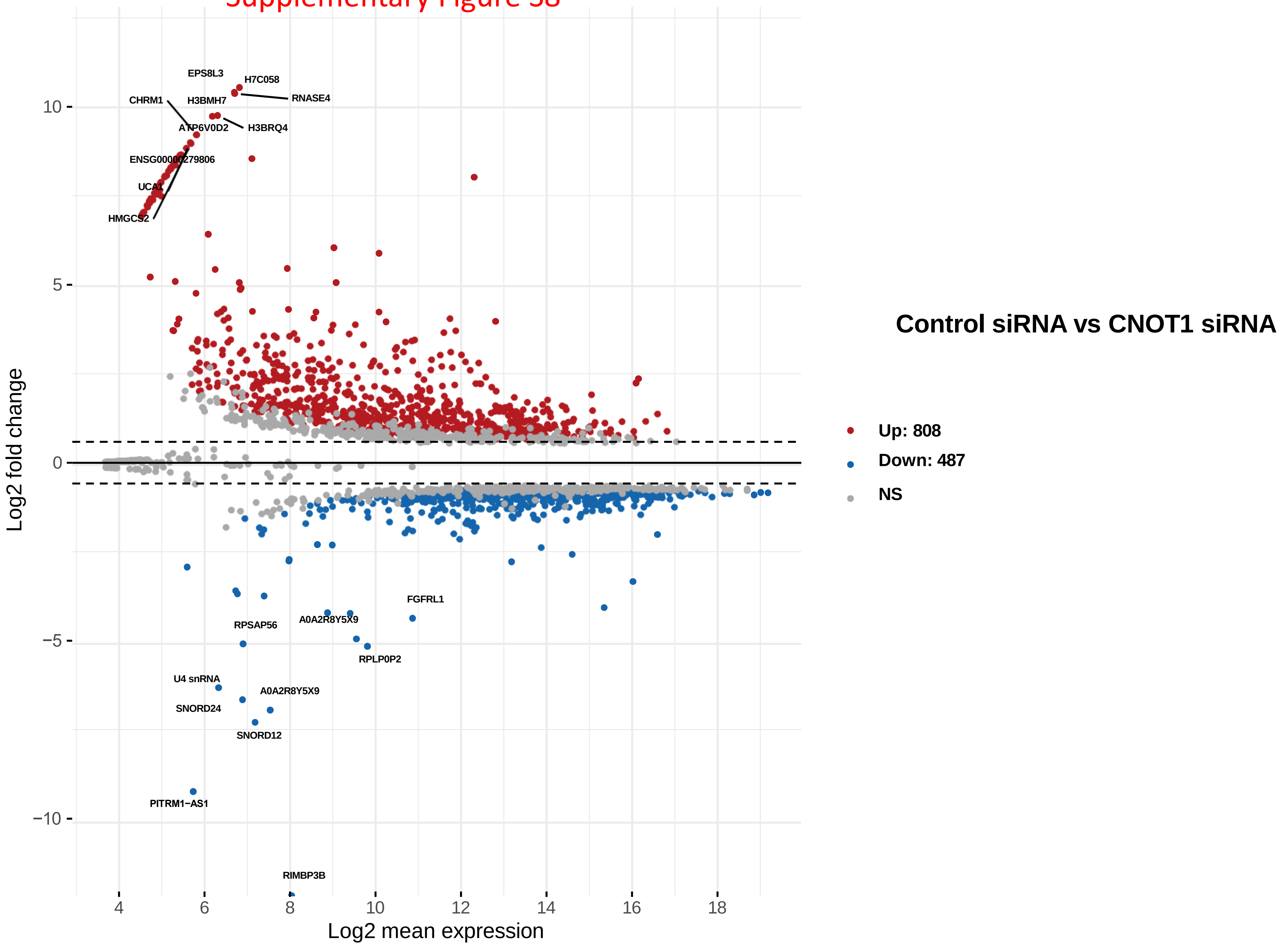

Supplementary Figure S9

A) Control siRNA

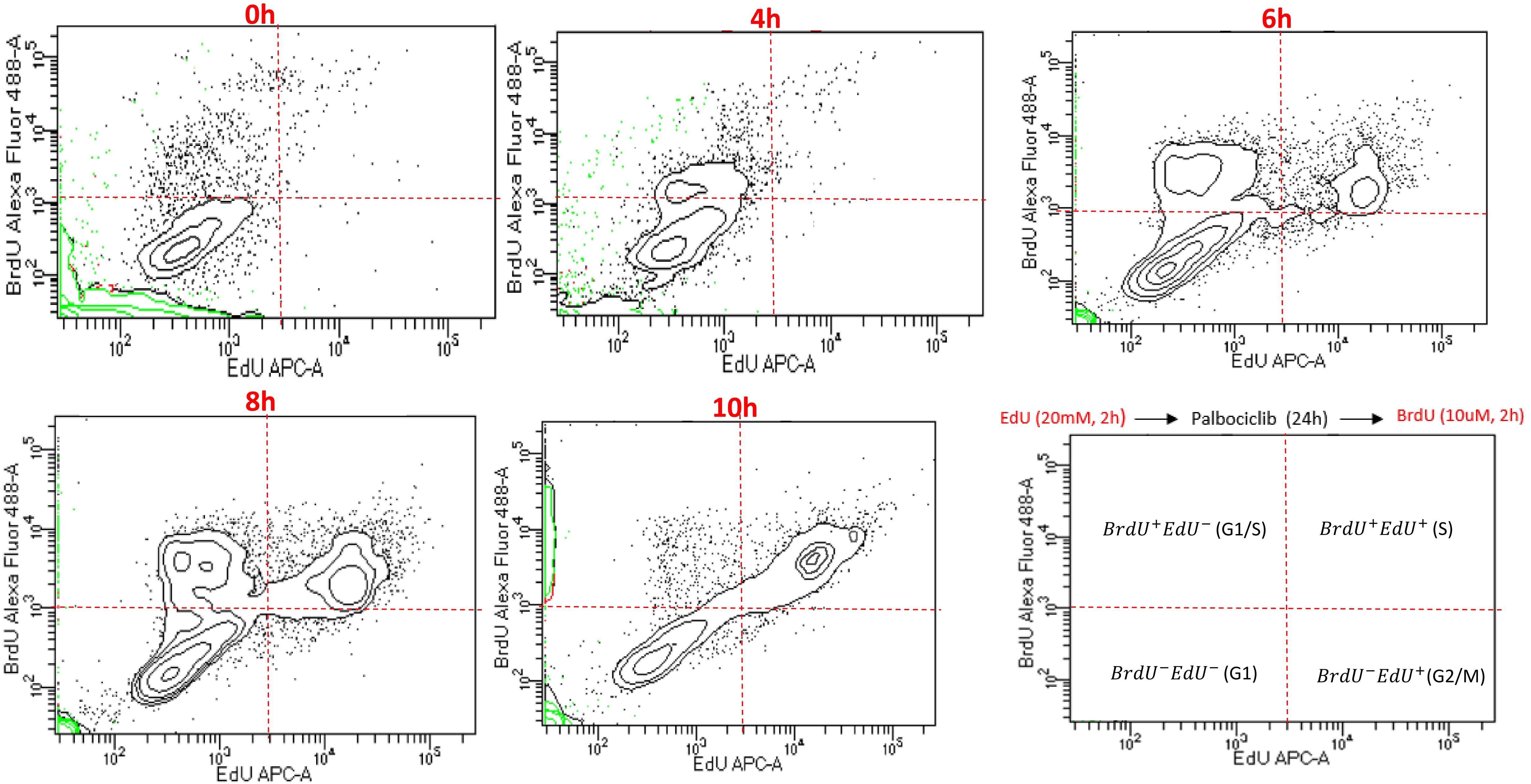

Supplementary Figure S9

B) CNOT1 siRNA

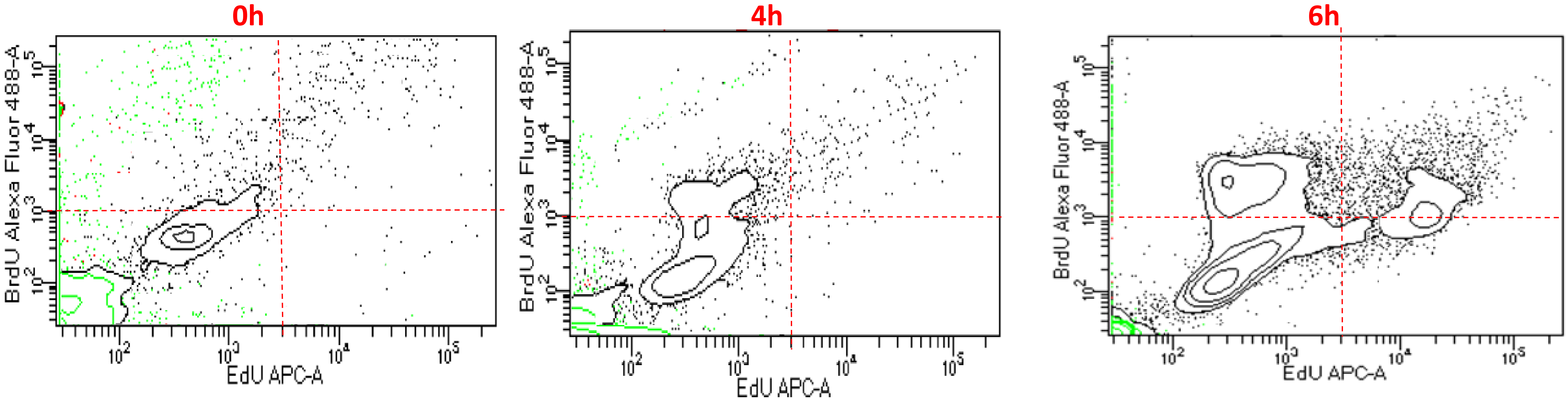

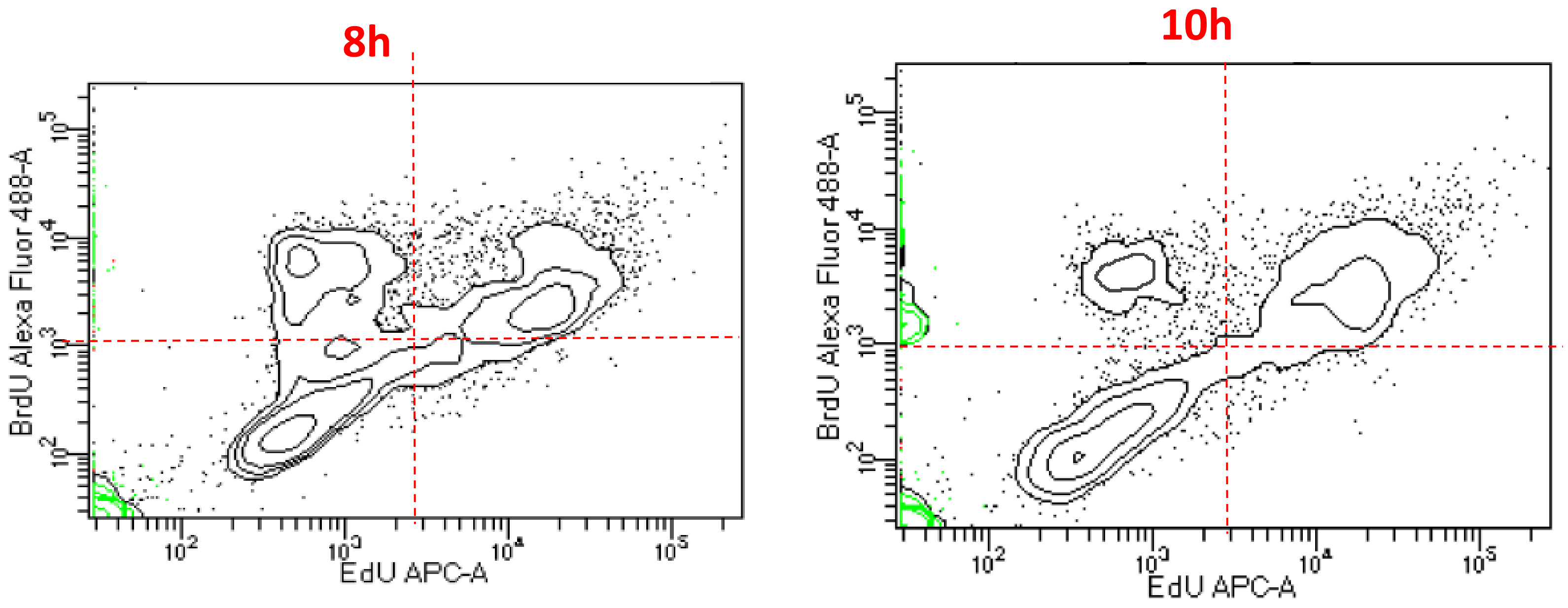

C)

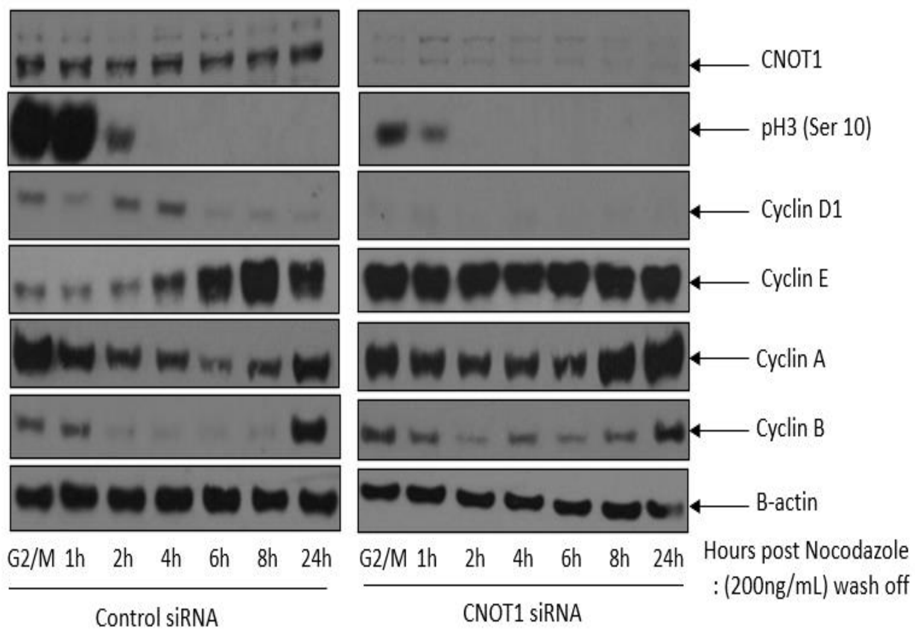

Supplementary Figure S10

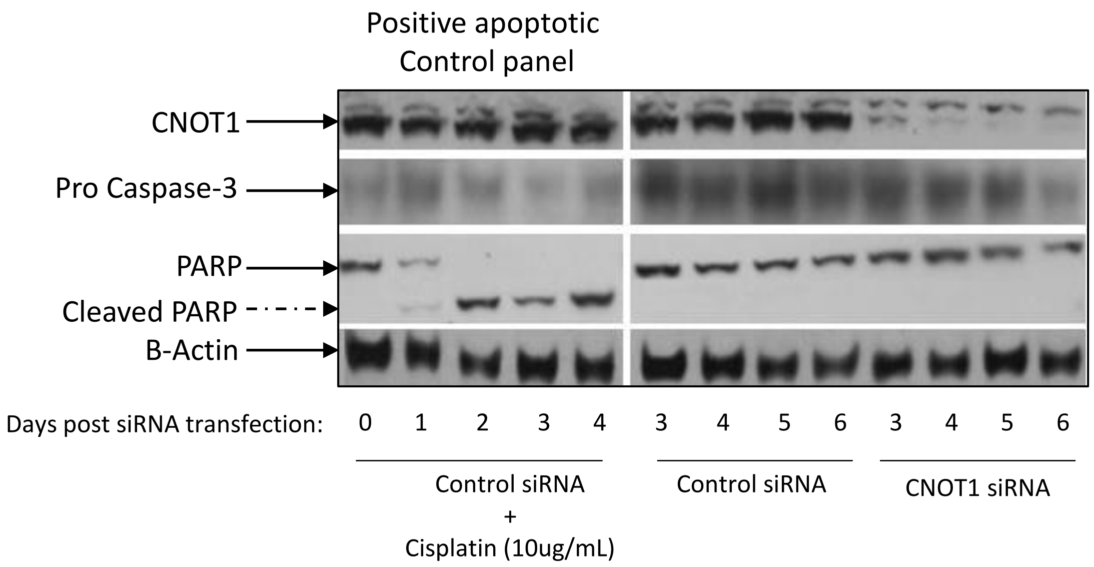

Supplement: Supplementary file 1 [file cells-12-01868-s001.zip › cells-1884880-supplementary.pdf]
